# Novel TRPM3 missense mutation leading to severe hypocalcemia presenting as seizures and complicated by non-sustained ventricular tachycardia: A case report

**DOI:** 10.5339/qmj.2025.28

**Published:** 2025-03-04

**Authors:** Pyrus Bhellum, Shekhar Angirekula, Amit Kumar Rohila, Ankur Sharma, Ankur Gupta, Namrata Mathur

**Affiliations:** ^1^Department of Internal Medicine, AIIMS, Jodhpur, India; ^2^Department of Trauma and Emergency, AIIMS, Jodhpur, India *Correspondence: Pyrus Bhellum. Email: pyrusbhellum@gmail.com

**Keywords:** Hypocalcemia, TRPM3, ventricular tachycardia, seizure, cardiomyopathy

## Abstract

**Background:**

Hypocalcemia is an electrolyte disorder that can be effectively corrected. However, in its severe form, it poses significant risks, including potentially fatal symptoms such as electrocardiographic changes that may lead to sudden cardiac arrest if not treated promptly.

**Case presentation:**

We report the case of a young female patient who presented with multiple episodes of tonic posturing and altered level of consciousness. Diagnostic evaluation revealed severe hypocalcemia with hypomagnesemia, QT prolongation, and episodes of non-sustained ventricular tachycardia. The condition was managed with calcium and magnesium supplementation. Further investigations revealed a novel missense mutation in transient receptor potential melastatin 3 (TRPM3).

**Discussion:**

Hypocalcemic seizures are rare in adults and are typically associated with severe hypocalcemia and cardiovascular instability, including ventricular dysrhythmias. The differential diagnoses in this case included primary hypoparathyroidism, Bartter syndrome type 5 (CaSR (calcium-sensing receptor) mutation), Gitelman syndrome, and claudin mutations. TRPM3 is highly expressed in kidney tissue, playing a role in the resorption of calcium and divalent ions. However, further research is needed to confirm its role in calcium homeostasis.

**Conclusion:**

The patient was initially misdiagnosed with epilepsy for the past two years. Following a comprehensive evaluation, she was successfully treated with intravenous calcium and magnesium. On follow-up after six months, her condition showed marked improvement, characterized by better cardiac function and the absence of further seizure episodes. This case represents the first reported instance of a TRPM3 mutation affecting calcium channels, highlighting the need for further investigation into its implications for calcium metabolism.

## Introduction

Calcium is a critical electrolyte with essential intracellular and extracellular functions, and its serum concentrations are meticulously maintained within a narrow physiological range.^1^ The major factors determining calcium homeostasis are parathyroid hormone (PTH), ionized calcium, calcitriol, vitamin D, and serum phosphate levels.^2^ The predominant clinical symptoms and signs of hypocalcemia are related to neuromuscular irritability, which include perioral paresthesia, tingling sensations in the fingers and toes, and involuntary muscle contractions. Additionally, hypocalcemia may lead to prolonged QT intervals, marked QRS complex, and ST-segment changes on the electrocardiogram, which can mimic acute myocardial infarction or conduction abnormalities.^3^ Ventricular arrhythmias are a rare complication of hypocalcemia. They are often associated with congenital long QT syndromes and other electrolyte abnormalities such as hypokalemia and hypomagnesemia, which often overlap with each other.^4,5^ The TRPM (transient receptor potential melastatin) family belongs to the superfamily of TRP cation channels.^6^ The TRPM subfamily is composed of eight members that play significant roles in diverse biological functions such as temperature sensing, inflammation, and insulin secretion. Among these, transient receptor potential melastatin 3 (TRPM3) is a non-selective cation-permeable channel. Although the pathophysiological effects of TRPM3 mutations in humans remain largely unexplored, recent research has identified two missense mutations in TRPM3 that are associated with a neurodevelopmental disorder characterized by intellectual disability, hypotonia, and epilepsy.^7^ A well-characterized function of TRPM3 in the literature pertains to its role in thermal nociception.^8^ However, the effects of these mutations on the functionality of calcium channels have yet to be investigated. TRPM 6 and 7 have been reported to be associated with hypomagnesemia and hypocalcemia known as hereditary hypomagnesemia with secondary hypocalcemia.^9,10^ Here, we report a case of a young female patient who arrived at our tertiary care center in Western Rajasthan, India, presenting with multiple episodes of tonic posturing and altered sensorium. Informed written consent was obtained from the patient.

## Case Report

A 25-year-old female with no known comorbidities presented to the Emergency Department at our tertiary care center in Western Rajasthan, India. She presented with complaints of multiple episodes of tonic posturing of bilateral upper and lower limbs over the past 15 days and an altered level of consciousness for the previous 10 days. She had a history of similar episodes for the past two years with 2–3 episodes every month, and was misdiagnosed as epilepsy. The frequency of these episodes increased to 3–4 episodes per day over the past 15 days. Each episode lasted for 30–45 seconds and was characterized by up-rolling of eyes, tongue biting, urinary incontinence, and postictal confusion lasting approximately 15–20 minutes. Additionally, the patient had intermittent tingling and numbness in all four limbs for the past two years. There was no history of chest pain, syncopal attacks, limb weakness, vomiting, abdominal pain, or decreased urine output. The patient was not on any antiepileptic drugs. Her father had a history of similar episodes of tonic contractions and numbness in all four limbs. He was diagnosed with hypocalcemia without any further etiological evaluation.

**Figure 1.**
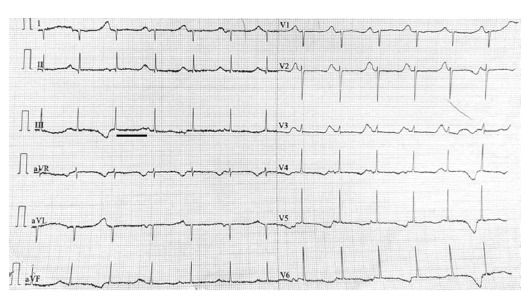
Electrocardiogram at presentation showing a prolonged QT interval, with Bazett's corrected QT interval of 861 ms (black line).

**Figure 2.**
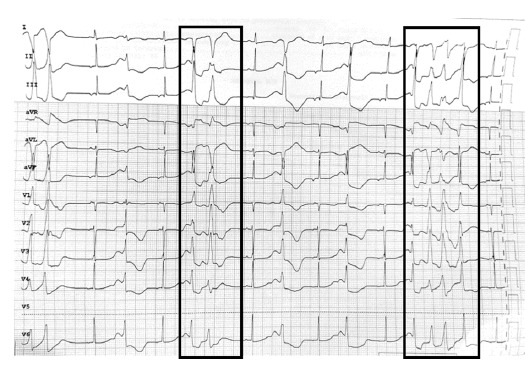
Electrocardiogram after one hour of presentation showing the presence of non-sustained ventricular tachycardia (black boxes).

At the presentation to our institute, she had active episodes of tonic movements in all four limbs. On examination, she appeared agitated but remained oriented to time, place, and person. The electrocardiogram revealed a prolonged QT interval, with Bazett's corrected QT interval of 861 ms (Figure 1). It showed intermittent non-sustained ventricular tachycardia (Figure 2).

**Figure 3.**
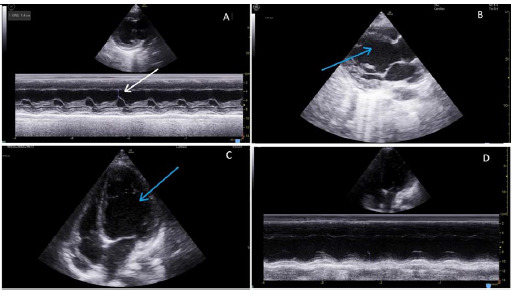
Two-dimensional echocardiographic (2D Echo) findings showing a high E-point septal separation (EPSS) of 1.4 cm (white arrow) (A), a dilated left ventricle (blue arrows) (B, C), and a reduced mitral annular plane systolic excursion (MAPSE) of 8 mm, all of which are suggestive of severe left ventricular systolic dysfunction (D).

On examination, her blood pressure was 94/60 mmHg, with a heart rate of 112 beats/minute. Blood gas analysis showed the presence of lactic acidosis with a low ionized calcium value of 0.47 mmol/L (1.16–1.31 mmol/L). Cardiac auscultation revealed loud P2 and no audible murmur. Other systemic examinations yielded no significant findings. A two-dimensional echocardiogram showed a dilated left ventricle (LV) with an ejection fraction (EF) of 25% and mild mitral regurgitation (Figure 3).

The levels of N-terminal pro B-type natriuretic peptide (NT pro BNP) were high at 5,056 ng/dl, indicating potential heart failure. Routine blood investigations revealed mild anemia, while total leukocyte and platelet counts remained within normal limits. Serum electrolyte analysis showed hypokalemia (K – 2.9 mg/dl), hypocalcemia (Ca – 2.3 mg/dl), hypomagnesemia (Mg – 1.3 mg/dl), and hyperphosphatemia (PO_4_ – 7.9 mg/dl). The level of intact parathormone was found to be low (4 ng/dl). The 24-hour urinary calcium level was 309 mg/day (normal: < 250 mg/day), indicating a potential renal tubular loss. Relevant laboratory investigations are presented in Table 1. Treatment involved the administration of one ampoule containing 10 ml of 10% calcium gluconate, which was diluted in 50 ml of 5% dextrose and infused over a period of 10 minutes. This was followed by an infusion of five ampoules of calcium gluconate in 5% dextrose over the next 10 hours. Additionally, a stat dose of 2 g of intravenous magnesium sulfate was administered in 100 ml normal saline over 30 minutes, along with potassium correction for hypokalemia. Due to persistent non-sustained ventricular tachycardia and the need for continuous cardiac monitoring, the patient was shifted to the intensive care unit after six hours of presentation. On day 2 of hospitalization, she developed cardiogenic shock and was on norepinephrine infusion for three days. Magnetic resonance imaging (MRI) of the brain revealed tortuosity and distension of the optic nerve, suggestive of chronic intracranial hypertension. Ocular examination revealed diffuse bilateral posterior subcapsular cataracts, while a dental examination revealed fluorotic changes and fractures in the third and fourth molars. Collectively, these findings were suggestive of chronic hypocalcemia. Additionally, cardiac MRI showed left ventricular global hypokinesia, with subtle segmental thinning and reduced systolic function (EF = 14%), along with mild pericardial effusion. Whole exome sequencing revealed a heterozygous missense variant in exon 10 of the TRPM3 gene (chr9:g.70640612T>C), resulting in the substitution of serine for asparagine at codon 465. A comprehensive family history revealed episodes of bilateral limb tightness in the patient's father, although no documentation was available to support the underlying etiology. Sequencing of the parents was recommended. However, it could not be performed due to the demise of both individuals.

Following a regimen of continuous intravenous infusions of calcium and magnesium, overlapping with an oral intake of 2 g/day of calcium with calcitriol and 25 mg hydrochlorothiazide/day, the patient exhibited symptomatic improvement after four days and shifted to oral medications exclusively. Her sensorium improved and also the episodes of ventricular tachycardia diminished. After a hospital stay of 32 days, she was discharged with stable vital signs on oral calcium carbonate at a dosage of 1,000 mg three times a day, oral magnesium oxide at 800 mg twice daily, tablet calcitriol at 0.25 mg once daily, and tablet hydrochlorothiazide at 12.5 mg once daily. Since her discharge, she had been on regular follow-up with no presenting symptoms. A repeat 2D echo conducted three months after discharge showed good LV systolic function, with an EF of 50–55% and a dilated LV.

## Discussion

Calcium homeostasis in the body is tightly regulated by PTH at the levels of the liver, kidneys, and gastrointestinal system.^11^ The secretion of PTH is strictly controlled by the levels of ionized serum calcium through a negative feedback mechanism, facilitated by the activation of calcium-sensing receptors (CaSR) mainly expressed on the surface of the parathyroid cells. Severe hypocalcemia in clinical settings is rare, with symptoms ranging from asymptomatic presentations to life-threatening ventricular dysrthythmias.^12^ Chronic hypocalcemia is usually asymptomatic and needs only oral and nutritional supplementation, while acute hypocalcemia can lead to neuromuscular symptoms and cardiovascular instability. Hypocalcemic seizures, which are rarely present in adults, are often associated with severe hypocalcemia and concomitant cardiovascular instability.^13^ Low levels of ionized calcium in the cerebrospinal fluid are associated with increased excitability in the central nervous system.^14^ When a patient presents with seizure episodes, it is essential to measure serum calcium levels to rule out hypocalcemia, along with assessing other electrolyte imbalances such as sodium and magnesium, as these can be reversible causes of seizures.^15^ Hypocalcemia causes prolongation of phase 2 (plateau phase) of the cardiac action potential, resulting in prolonged opening of L-type calcium ionic channels, allowing late calcium inflow and early depolarization. New action potentials are generated once the depolarization threshold is reached, which can trigger re-entry and tachyarrhythmias, eventually leading to ventricular tachycardia, ventricular fibrillation, and torsades de pointes.^16^ The QT prolongation correlates with the severity of hypocalcemia. Most often, hypocalcemia is associated with concomitant hypokalemia and hypomagnesemia, which may exacerbate the electrocardiographic (ECG) changes. Cardiac manifestations can vary significantly, ranging from giddiness and syncope to life-threatening complications such as dysrhythmias, cardiomyopathy, and heart failure.^17,18^ Hypocalcemia-induced heart failure responds very slowly to diuretics, sometimes showing resistance, but may become reversible after prolonged therapy.^19,20^ In the case presented, the patient had an EF of 25%, with cardiogenic shock, which gradually improved after calcium correction. The differential diagnoses in our case were primary hypoparathyroidism, Bartter syndrome type 5 (CaSR mutation), Gitelman syndrome, and claudin mutations involving the paracellular pathway of magnesium and calcium at the thick ascending limb of the loop of Henle. Bartter syndrome was considered less likely due to the presence of hypomagnesemia, and the possibility of Gitelman syndrome was also low given the presence of hypercalciuria instead of hypocalciuria.^21^ Ultrasonography of the abdomen was performed, revealing normal-sized kidneys without signs of nephrocalcinosis. This finding was inconsistent with the diagnosis of claudin mutations, as significant nephrocalcinosis, which can lead to end-stage renal disease, is well documented in patients with claudin mutations.^22^ Therefore, our differential diagnoses were narrowed down to CaSR mutation and primary hypoparathyroidism, both of which could have overlapping features. Whole exome sequencing revealed a heterozygous missense variant in exon 10 of the TRPM3 gene (chr9:g.70640612T>c), resulting in the substitution of serine for asparagine at codon 465. Although TRPM3 is a non-selective cation permeable channel, mutations in this gene have not yet been reported to cause effects on calcium homeostasis or metabolism.^7^ Furthermore, two missense mutations in TRPM3 have been associated with neurodevelopmental disorder characterized by intellectual disability, hypotonia, and epilepsy.^7^ A well-characterized function of TRPM3 in the literature pertains to its role in thermal nociception.^8^ This report represents the first instance of a TRPM3 mutation impacting calcium channel function. Notably, kidney tissue has high expression levels of TRPM3, particularly in the medullary ray, the renal corpuscle, and the epithelium of the collecting tubule, suggesting its potential role in the resorption of calcium and other divalent ions.^6,23^ This hypothesis explains the hypercalciuria, hypocalcemia, and hypomagnesemia observed in our patient. However, it does not provide definitive evidence regarding the role of TRPM3 in the homeostasis of calcium or other divalent ions. Further research is warranted to explore the effects of TRPM3 mutations on calcium metabolism.

The clinical manifestations of hypocalcemia and hypoparathyroidism are complex and may be misdiagnosed as primary epilepsy. In our case, the patient was misdiagnosed with “epilepsy” for almost two years, during which a thorough evaluation was not conducted to rule out secondary causes. The ECG also revealed QT prolongation, leading to intermittent episodes of non-sustained VT, which could have been potentially lethal for the patient. Therefore, it is important to investigate calcium levels in patients presenting with cardiac dysrhythmias, ECG abnormalities, and seizure episodes, along with the timely management of any abnormalities to prevent life-threatening emergencies.

## Conclusion

This case highlights the critical need for the prompt recognition and management of severe hypocalcemia, a condition that can be potentially life-threatening and can present with cardiovascular instability and neurological manifestations. The patient was initially misdiagnosed with epilepsy for two years, during which severe hypocalcemia, hypomagnesemia, QT prolongation, and episodes of non-sustained ventricular tachycardia were identified. Timely intervention with intravenous administration of calcium and magnesium resulted in clinical stabilization, with no recurrence of seizures and improved cardiac function observed at six-month follow-up. Genetic analysis revealed a novel TRPM3 mutation, suggesting its potential role in calcium homeostasis, a mechanism that remains inadequately understood in existing literature. This case highlights the need to consider electrolyte abnormalities in the differential diagnosis of seizure disorders. Furthermore, there is a critical need for additional research to elucidate the role of TRPM3 in calcium homeostasis and its pathophysiological significance. Early correction of electrolyte imbalances is of paramount importance in preventing complications and optimizing patient outcomes.

### List of abbreviations

[Table tbl2]

### Competing interests

The authors have no conflicts of interest to declare.

## Figures and Tables

**Table 1.** Relevant laboratory investigations.

**Laboratory parameters**	**At presentation**	**Reference values**

Hemoglobin	10.9 g/dl	12–15 g/dl

Total leukocyte counts	10.89(10^3^/mL	(4–11) × 10^3^/mL

Neutrophils/lymphocytes/monocytes	60.8/18.5/8.6%	40–60/20–40/2–8%

Platelets	211 × 10^3^/uL	150–400(10^3^/uL

AST	65.8 IU/L	< 35 IU/L

ALT	35.2 IU/L	< 35 IU/L

ALP	210 IU/L	40–120 IU/L

Albumin	3.39 g/dl	3.5–5.2 g/dl

Serum urea	23 mg/dl	17–43 mg/dl

Serum creatinine	1.39 mg/dl	0.66–1.09 mg/dl

Sodium	135 mEq/L	135–145 mEq/L

Potassium	2.98 mEq/L	3.5–5.0 mEq/L

Chloride	86 mEq/L	101–109 mEq/L

Calcium	2.81 mg/dl	8.6–10.3 mg/dl

Magnesium	1.32 mg/dl	1.9–2.5 mg/dl

Phosphorus	7.53 mg/dl	2.5–4.5 mg/dl

Instant PTH	4.8 pg/mL	11.7–61.1 pg/ml

Vitamin D	10.0 ng/ml	30–100 ng/ml

8 a.m. Cortisol	19.78 mcg/dl	4.3–22.4 mcg/dl

LDH	462 U/L	< 248 U/L

TSH	1.548 mIU/L	0.3–3.6 mIU/L

fT3	2.162 pg/mL	2.2–4.2 pg/mL

fT4	1.926 ng/dL	0.8–1.7 ng/dL

Anti-TPO	2.168 U/mL	< 60 U/dL

Ferritin	415.3 ng/ml	10–291 ng/ml

24-hour urine calcium	309.96 mg/day	< 250 mg/day

24-hour urine magnesium	213 mg/day	72–122 mg/day

Plasma osmolality	278 mOsm/kg	275–300 mOsm/kg

Urinary osmolality	206 mOsm/kg	500-850 mOsm/kg

Urinary creatinine	11.17 mg/dl	10–200 mg/dl

Urinary albumin creatinine ratio	71 mg/g	< 30 mg/g

NT pro BNP	5,056 pg/ml	< 125 pg/ml

MRI brain	There is vertical tortuosity of the bilateral optic nerve with enlargement of the subarachnoid space surrounding the optic nerves with a flattening of the posterior sclera, a partially empty sella, and bilateral enlargement of the Meckel cave – features suggestive of intracranial hypertension. Rest of the brain parenchyma shows a normal differentiation between gray and white matter. No abnormal signal intensity is observed. There is no restricted diffusion or increased susceptibility. Brainstem, cerebellum, basal ganglia, and thalami present normal signal intensity. Major arterial and venous flow voids are normally observed. Ventricular system is normal.	

Cardiac MRI	General: The cardiac chambers exhibit normal viscero-atrial, atrio-ventricular, and ventriculo-arterial concordance.	

	Left ventricle: The left ventricle (LV) shows global hypokinesia. There is subtle thinning of the respective segments with reduced systolic function. LV EF=14%. Late gadolinium enhancement:	

	Focal linear enhancement (involving up to 50% of myocardial thickness) is observed in the intramural region along the interventricular septum with wall motion abnormalities as detailed above. No evidence of LV apical thrombus on cine or LGE images. Right ventricle: The right ventricle is normal in size and shape and shows normal systolic function. No evidence of right ventricular wall or trabecular hypertrophy is noted. Left atrium: The left atrium is normal. No evidence of thrombus is noted in the left atrial appendage. Right atrium: The right atrium is normal in size. Interatrial septum: The interatrial septum is intact and normal in thickness. Valves: The aortic, pulmonary, mitral, and tricuspid valves appear grossly normal. Pericardium: The pericardial thickness appears normal. No evidence of significant post-contrast pericardial enhancement is noted. Mild pericardial effusion is present. Great vessels: The visualized thoracic aorta and pulmonary arteries appear grossly normal. Both the SVC and IVC are normal in caliber.	


**Table tbl2:** 

ALP	Alkaline Phosphatase

ALT	Alanine Aminotransferase

Anti-TPO	Anti-Thyroid Peroxidase Antibodies

AST	Aspartate Aminotransferase

EF	Ejection Fraction

fT3	Free Triiodothyronine

fT4	Free Thyroxine

IVC	Inferior Vena Cava

LDH	Lactate Dehydrogenase

LGE	Late Gadolinium Enhancement

LV	Left Ventricle

MRI	Magnetic Resonance Imaging

NT pro BNP	N-terminal pro B-type Natriuretic Peptide

SVC	Superior Vena Cava

TSH	Thyroid-Stimulating Hormone

